# Intrinsic motivation learning for real robot applications

**DOI:** 10.3389/frobt.2023.1102438

**Published:** 2023-02-10

**Authors:** Rania Rayyes

**Affiliations:** Institut für Fördertechnik und Logistiksysteme, Karlsruher Institut für Technologie, Karlsruhe, Germany

**Keywords:** intrinsic motivation (IM), lifelong learnig, mental replay, goal directed learning, open-ended learning, active learning

## 1 Introduction

Humanoid robots are built to resemble the human body and mimic human motion and interaction (Hirai et al., 1998; Tikhanoff et al., 2010; Kajita et al., 2014). The recent research in this field aims to integrate these robots in our daily life, e.g., collaborative robots (Asfour et al., 2019; Ogenyi et al., 2021), social robots (Sandini et al., 2018), and service robots (Van Pinxteren et al., 2019). However, integrating such robots in our daily life is challenging because pre-programmed tasks and traditional control methods restrict the robots’ adaptability and flexibility. This shifts the research focus toward new machine learning methods for lifelong learning which enable autonomous online adaptation and continuous data-driven learning (Nguyen and Oudeyer, 2014; Parisi et al., 2019). Since humanoid’s design is closely related to humans, it is, therefore, essential to incorporate cognitive capabilities, learning skills and human-like abilities, e.g., curiosity and self-learning in these robots.

Recent developments in robotics and cognitive science may lead to a new generation of more versatile and adaptive robots, named Developmental Robots (Asada et al., 2001; Lungarella et al., 2003). Developmental robotics is a highly interdisciplinary research field linking natural and artificial systems. On the one hand, it aims to develop learning approaches for humanoids inspired by developmental aspects and learning mechanisms observed in children (Kim et al., 2008; Asada et al., 2009; Cangelosi et al., 2015). On the other hand, humanoids also serve as experimental platforms for better understanding of biological development (Asada et al., 2001; Asada et al., 2009; Cangelosi et al., 2015; Asano et al., 2017).

Developmental robots must autonomously develop, adapt and acquire their skills through their life-time, i.e., lifelong learning (Lungarella et al., 2003; Mai, 2013; Forestier, 2019). In contrast to industrial robots, which accomplish predefined tasks, developmental robots must solve unpredictable tasks, learn new skills, adapt to new environments, and cope with unforeseen challenges. Intrinsic motivation methods tackle these challenges through driving the robot’s learning and exploration autonomously by internally generated signals in an open-ended (i.e., unbounded) environment (Schmidhuber, 2010; Baranes and Oudeyer, 2013; Santucci et al., 2016; Baldassarre, 2019; Rayyes et al., 2020a; Rayyes, 2020; Rayyes et al., 2021). However, the high sample-complexity of these methods, i.e., the dense sampling required to approximate the learned function with a reasonable accuracy, restrict their real-world applications. Therefore, the majority of previous work has been demonstrated only in simulation as a proof of concept, and only a few were demonstrated in real robot experiments, e.g., (Tanneberg et al., 2018; Huang et al., 2019; Rayyes et al., 2020a; Rayyes et al., 2021).

In my opinion, increasing the sample-efficiency and the applicability of the intrinsic motivation methods can be done by combining them with mental replay (Andrychowicz et al., 2017; Rayyes et al., 2020b) and goal-directed methods, e.g., Goal Babbling (Rolf et al., 2011), as shown in the literature so far.

## 2 Intrinsic motivation

Intrinsic motivation in robotics has been inspired by developmental psychology, in which curiosity-driven behavior has been observed in children. Children get easily bored by known items and seek new ones driven by their curiosity to improve their knowledge and gain new experience (Schmidhuber, 2010). Intrinsic motivation methods in the literature can be sorted into two categories (Oudeyer and Kaplan, 2007; Santucci et al., 2013; Forestier, 2019; Rayyes, 2020): 1) knowledge-based, where the intrinsic motivation signal is devised based on the error between the prediction of the robot and its real outcome; 2) competence-based, where the intrinsic motivation signal is devised based on the learning progress of the robot. However, an experiment in (Baranes et al., 2014) showed that humans learn by maximizing their knowledge of a task and their competence. Accordingly, a recent intrinsic motivation method named “Interest Measurement” (Rayyes et al., 2020b) combined both knowledge-based and competence-based signals.

### 2.1 Knowledge-based intrinsic motivation

The knowledge-based intrinsic motivation methods in the literature are either novelty-based or prediction-based (Barto et al., 2013; Baldassarre, 2019) Novelty-based learning refers to learning from novel information and the intrinsic motivation signal is generated by comparing newly acquired knowledge with previously gained one (Baldassarre, 2019; Forestier, 2019), e.g., comparing observed scenes (Huang and Weng, 2004) to guide the robot’s exploration to discover new ones. Other examples are the intrinsic motivation signal in (Benureau and Oudeyer, 2016), which maximizes the diversity of the robot’s behaviors, and the intrinsic motivation signal in (Frank et al., 2014), which maximizes information gain by comparing (action-state) distribution before and after learning update. In (Oudeyer and Kaplan, 2007), novelty is detected based on a specific error threshold (Oudeyer and Kaplan, 2007).

Prediction-based learning refers to learning from prediction errors of the robot (Forestier, 2019), where high prediction errors indicate a good opportunity to learn from (Chentanez et al., 2005; Zhang et al., 2014). For example, the learning signal in (Rayyes et al., 2020a) measures the error between the robot’s performance and the robot’s prediction for reaching objects. The higher the error is, the more interesting the object becomes. Other authors named a prediction-based intrinsic motivation as surprise (Oudeyer and Kaplan, 2007; Barto et al., 2013). Other examples are the penalty signal (Huang et al., 2019), which is a dynamics-based surprise signal to avoid applying high forces during learning, and Bayesian surprise (Storck et al., 1994), which is used as a curiosity reward. In contrast, the free energy principle (Schwartenbeck et al., 2013; Kaplan and Friston, 2018; Ahmadi and Tani, 2019) assumes that humans try to minimize the long-term average of surprise. Minimizing surprise leads to maximizing model-evidence for intrinsically motivated agents in the context of decision-making.

The difference between prediction-based and novelty-based signals has been experimentally investigated (Caligiore et al., 2015). The results showed that novelty-based signals were more effective to drive the human learning. Still, there is no clear border between these two categories since high prediction errors indicate novel situations to learn from as shown recently in the novelty detection method (Rayyes et al., 2021). Similarly, (Barto et al., 2004; Oudeyer and Kaplan, 2007) considered high prediction error as a novelty-based signal.

### 2.2 Competence-based intrinsic motivation

Competence-Based methods measure the robot’s performance over time instead of instantaneous measures of the prediction errors (Schmidhuber, 1991; Baranes and Oudeyer, 2013; Rayyes et al., 2020a). For example, (Baranes and Oudeyer, 2013) monitored the performance error over a sliding window during the robot’s exploration. The most interesting regions of the workspace for the robots are where the robot demonstrates high changes in the error prediction regardless whether the error increases or decreases. In other words, the robot’s exploration is guided through the intrinsic motivation signal toward the regions where the robot’s performance changes drastically, whether the robot’s performance enhancing (learning) or deteriorating (forgetting). The Learning progress method in (Santucci et al., 2016) considered only when the error decreases over a sliding window, i.e., when the robot learns. This method drives the robot’s explorations toward easily learn-able tasks and avoids to learn near the border of the workspace as shown in (Rayyes, 2020). However, the main advantage of this method is that, it can avoid unreachable/unlearn-able objects/tasks (Santucci et al., 2016). In contrast, the forgetting factor method in (Rayyes et al., 2020a) monitors if the robot’s performance deteriorates. This allows the robot to refocus on the previously learned forgotten experiences, which might happens in the lifelong learning (Rayyes, 2020) due to continuous model update.

Most recent intrinsic motivation methods are competence-based methods (Schmidhuber, 2010; Baranes and Oudeyer, 2013; Santucci et al., 2013; Nguyen and Oudeyer, 2014; Forestier and Oudeyer, 2016; Santucci et al., 2016). (Santucci et al., 2013) showed that competence-based methods often lead to better performance than knowledge-based ones. A comparison between the methods was demonstrated for learning several reaching tasks using a simulated robot manipulator. However, how to transfer these results to more complex real-world robot applications remains an open question.

### 2.3 Combining knowledge-based with competence-based intrinsic motivation

While most intrinsic motivation methods in the literature are either knowledge-based or competence-based, an experimental study (Baranes et al., 2014) showed that humans tend to learn by trying to improve their knowledge about the tasks in hand and their competence. Interest Measurement (Rayyes et al., 2020b) is a recent intrinsic motivation method which combines knowledge-based with competence-based signals. The knowledge-based signal, named relative error, drives the robot’s exploration toward difficult to attain goals/tasks, e.g., goals near the border of the robot’s workspace. The competence-based signal is the forgetting factor which monitors where the robot’s performance deteriorates during lifelong learning. This combination of different learning signals led to high sample-efficiency which facilitates online data-driven direct learning on real robots without any pre-training in simulation as shown in (Rayyes et al., 2020a; Rayyes et al., 2020b; Rayyes, 2020).

## 3 Intrinsic motivation in real applications

The main challenge for intrinsic motivation is the applicability due to the high sample-complexity of the proposed methods. Therefore, only a few methods have been demonstrated on real robots, e.g., (Oudeyer et al., 2007; Hart and Grupen, 2011; Duminy et al., 2016; Forestier et al., 2017; Tanneberg et al., 2018; Huang et al., 2019). However, not all of these methods have demonstrated efficient learning or goal-directed exploration. For instance, in (Forestier et al., 2017; Seepanomwan et al., 2017) the robot preformed random movements during the exploration which is inefficient and incompatible with humans’motion (von Hofsten, 2004). In contrast, (Tanneberg et al., 2018; Huang et al., 2019; Rayyes et al., 2021; 2020a) have demonstrated high sample-efficiency and goal-directed motion. The only methods with notable high sample-efficiency are the methods which integrated intrinsic motivation with mental replay methods (Andrychowicz et al., 2017; Rayyes et al., 2020b).

### 3.1 Mental replay

Mental replay is an essential component in human learning (Foster and Wilson, 2006). Mental replay methods have been proposed for robotics to reduce sampling complexity and to speed up the learning process (Lin, 1993; Mnih et al., 2013; Andrychowicz et al., 2017; Riedmiller et al., 2018; Tanneberg et al., 2018; Gerken and Spranger, 2019; Rayyes et al., 2020b). Therefore, these methods are essential for deploying data-driven learning methods on real robots, since sampling in real robot applications is very costly regarding time and hardware. Additionally, Mental Replay has been used to overcome forgetting in lifelong learning (Parisi et al., 2019).

## 4 Discussion

Intrinsic motivation is very promising to integrate humanoids in our daily life. It is compatible with online and lifelong learning, and it provides adaptability and flexibilities for the robots. Since the main challenge of intrinsic motivation methods are the high sample complexity for real robot applications due to tear and wear. The question is how to increase the potential of these methods to be applied in real-world scenarios. The only solution to pave the way for real robot applications is to increase the sample-efficiency. On the one hand, the mental replay methods play a significant role to decrease drastically the amount of required data to learn a model with a reasonable accuracy. On the other hand, the learning and the exploration should be organized as goal-directed motion, e.g., Goal Babbling (Rolf and Steil, 2014), active learning (Baranes and Oudeyer, 2013), interest-driven Goal Babbling (Rayyes et al., 2020b), etc. Random exploration to collect data is unrealistic for robots with many degrees of freedom. The respective high-dimensional spaces, e.g., for motor commands, cannot be exhausted through random or systematic exploration owing to a combinatorial explosion. Additionally, studies on infants have shown that neonates do not behave randomly, but rather demonstrate goal-directed motion a few days after birth (von Hofsten, 2004). Hence, combining purely goal directed methods with mental replay and intrinsic motivation can increase the sample-efficiency remarkably and accordingly can be deployed on real robots.
